# Facilitation of oral sensitivity by electrical stimulation of the faucial pillars

**DOI:** 10.1038/s41598-021-90262-y

**Published:** 2021-05-24

**Authors:** Tobias Braun, Samra Hamzic, Johanna M. Doerr, Laura Peters, Maxime Viard, Iris Reuter, Mario Prosiegel, Susanne Weber, Mesut Yenigün, Marlene Tschernatsch, Tibo Gerriets, Martin Juenemann

**Affiliations:** 1 Department of Neurology, University Hospital Giessen and Marburg, Justus Liebig University, Klinikstrasse 33, 35392 Giessen, Germany; 2 Faculty of Medicine, Justus-Liebig-University Giessen, Klinikstrasse 29, 35392 Giessen, Germany; 3 Department I, Faculty of Languages and Literatures, Ludwig-Maximilians-University (LMU), Munich, Germany; 4 Stroke Unit, Buergerhospital Friedberg, Ockstaedter Str. 3-5, 61169 Friedberg, Germany; 5 Department of Neurology, Gesundheitszentrum Wetterau, Chaumontplatz 1, 61231 Bad Nauheim, Germany

**Keywords:** Physiology, Neurology

## Abstract

Dysphagia is common in neurological disease. However, our understanding of swallowing and its central nervous control is limited. Sensory information plays a vital role in the initiation of the swallowing reflex and is often reduced in stroke patients. We hypothesized that the sensitivity threshold of the anterior faucial pillar could be facilitated by either electrical stimulation (ES) or taste and smell information. The sensitivity threshold was measured by ES in the anterior faucial pillar region. The measurement was repeated 5 min after baseline. Thirty minutes after baseline, the participants underwent a test for taste and smell. Immediately after the test, the ES was repeated. Thirty healthy volunteers with a mean age of 27 ± 5.1 participated in the trial. Mean sensitivity threshold at baseline was 1.9 ± 0.59 mA. The values 5 min after baseline (1.74 ± 0.56 mA, *p* = 0.027) and 30 min after baseline (1.67 ± 0.58 mA, *p* = 0.011) were significantly lower compared to the baseline, but there was no difference between the latter (*p* = 0.321). After 5 min, a potentially facilitating effect was found on oral sensitivity by ES of the faucial pillar area. Thirty minutes later, this effect was still present.

**Trial registration** Clinicaltrials.gov, NCT03240965. Registered 7th August 2017—https://clinicaltrials.gov/ct2/show/NCT03240965**.**

## Introduction

Dysphagia is encountered in various diseases and clinical specialties, especially in neurological patients, and has gained in scientific importance recently. It is associated with increased mortality, morbidity, a longer hospital stay, and high treatment costs^1–3^. In the acute phase of stroke, up to 80% of patients are diagnosed with swallowing disorders^4^. The most serious consequence of dysphagia after stroke is aspiration pneumonia, being the most common cause of death in stroke patients^5^. Therefore, dysphagia determines the immediate prognosis and is of particular relevance to neurological patients due to the functional link to the central and peripheral nervous system.

Although research in the field of dysphagia is increasing, there are still large gaps in our understanding of swallowing and its central nervous control.

Scientific literature on sensory functions in normal and disordered swallowing is heterogeneous. It differs on the importance or unimportance in the swallowing process of various oropharyngeal regions such as mucosa of the tongue base, faucial pillars, valleculae, epiglottis, or laryngeal folds. The heterogeneity stems from various experiments on human subjects (healthy controls or patients) or animals (rats, lambs, etc.) and different methods for measurement of sensory function or mode of swallowing (voluntary vs. involuntary)^6^. The mode of swallowing in particular is thought to have a significant effect on the swallowing process due to the fact that voluntary swallowing appears to be entirely different from involuntary swallowing^7–10^.

The hallmark of stroke-related dysphagia is loss of oropharyngeal sensitivity leading to uncontrolled leaking of the bolus from the oral cavity into the pharynx and a delayed triggering of the swallowing reflex, predisposing the patient to penetration of the bolus to the larynx and aspiration into the trachea without sufficient protective reflexes^11–14^.

Various approaches to assess oral sensory function already exist. Stimulation of oropharyngeal regions using air puffs, testing with Frey filaments, or simple bilateral testing using a cotton swab were described earlier^15,16^. An objective and quantifiable approach was first described by Power et al. using electrical stimulation (ES) of oral structures with an electrode mounted on a gloved finger^17^. Apart from the cotton swab, no method has been integrated into clinical routine.

To assess sensory function in a large cohort of stroke patients using the method described by Power et al., it was necessary to first generate data from healthy controls. The sensory threshold was examined in a specific region of the oral cavity, namely the anterior faucial pillar.

The faucial pillar region is of particular interest for our research, being the most distal area of the oropharyngeal tract that can be reached easily with the examiner’s index finger and without any side effects such as coughing or gagging. It is assumed that the faucial area plays a significant role in swallowing. Power et al. identified projections via the glossopharyngeal nerve from each faucial pillar to the sensory cortex of both hemispheres with ipsilateral predominance^18^. Data suggest that the contact of the bolus with the faucial pillar triggers the swallowing reflex^19–22^, although some authors found no evidence for this theory^23,24^. This theory stems from the observation that the elevation of the hyoid, which marks the time of pharyngeal swallowing, begins when the bolus makes contact with the faucial pillar region^25,26^.

ES of oral and pharyngeal regions has been implemented as a therapeutic approach to improve swallowing function. This is thought to activate neuroplasticity via sensory pathways^27,28^. Stimulation with a frequency of 5 Hz over 10 min over 3 days was found to have the largest effect^29,30^ on swallowing function.

We therefore hypothesized that the examination of oral structures via ES itself might facilitate the sensory function by lowering the sensory threshold, although we planned to use different parameters for stimulation (see below). A facilitating effect would be important for further trials, as this might change the results depending on the point of time or repetition of testing. To test our hypothesis, we repeated the examination of the bilateral sensory threshold in our participants to evaluate changes evoked by the stimulation itself.

As described in previous works, latency of swallowing reflex is reduced when olfactory and gustatory information is presented^6,31–33^. For this reason, the influence of taste or smell information on sensory function was investigated in our trial as well.

## Results

### Participants’ characteristics

The median age in the group of volunteers was 27 ± 5.1, 16 persons (53.3%) were male, 2 (6.7%) were left-handed, and 5 (16.7%) reported cigarette smoking. The mean taste score was 11.83 ± 1.86, and the mean smell score was 11.13 ± 0.86.

### Sensitivity threshold testing

In one person, we were unable to measure the sensitivity threshold of the left side, even when we increased the intensity to 100 mA.

A repeated-measures ANOVA showed a significant effect of time (F(1.839, 53.323) = 5.039; *p* = 0.012; Greenhouse–Geisser).

**Figure 1:**
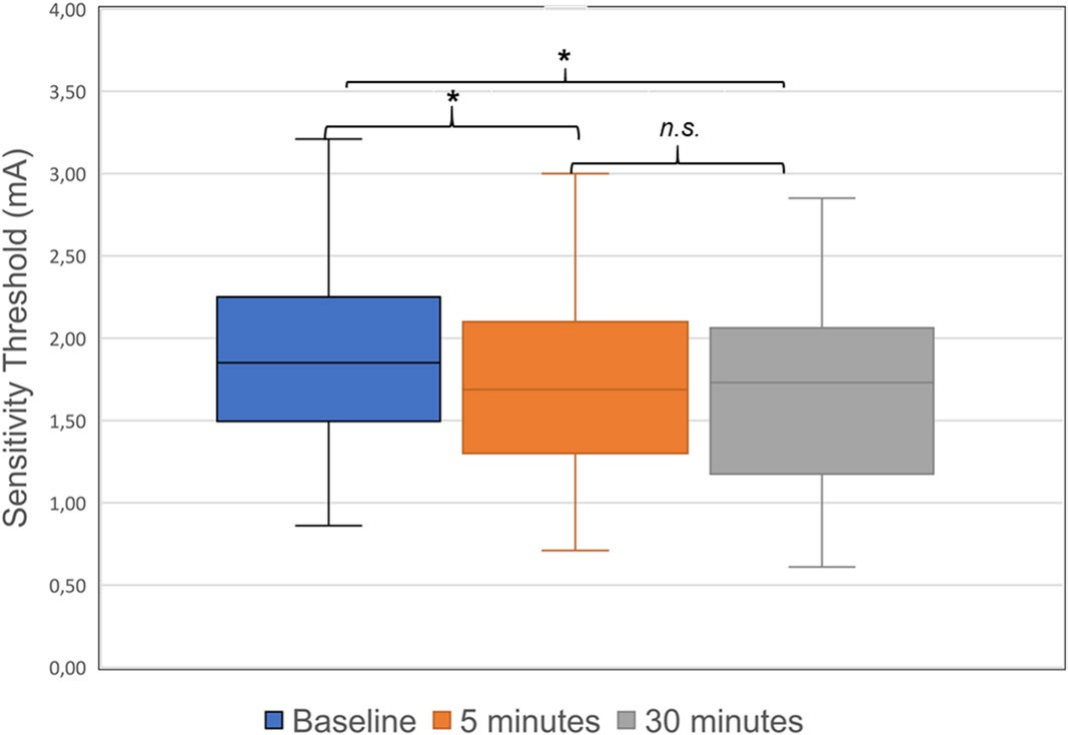
Measurements of sensitivity threshold presented as mean and standard deviation. The values 5 min after baseline (1.74 ± 0.56 mA, *p* = 0.027, Bonferroni corrected *p* = 0.081) and 30 min after baseline (1.67 ± 0.58 mA; *p* = 0.011, Bonferroni corrected *p* = 0.033) are significantly lower compared to baseline, but there is no difference between 5 and 30 min after baseline (*p* = 0.321, Bonferroni corrected *p* = 0.963).

The sensitivity threshold was lower compared to baseline 5 min after the first measurement (1.9 ± 0.59 mA vs. 1.74 ± 0.56 mA; t(29) = 2.321; *p* = 0.027; Cohen’s d = 0.42; Bonferroni corrected *p* = 0.081). As this effect (although only significant by trend when corrected for multiple comparisons) could have been due to a training effect, we analysed the three values of the baseline measurement. The values for each individual side and the total of both sides showed high consistency (ICC_left_ = 0.75, ICC_right_ = 0.66) and did not differ statistically (*p* > 0.05). This effect was also present after 30 min (1.9 ± 0.59 mA vs. 1.67 ± 0.58; t(29) = 2.721; *p* = 0.011; Cohen’s d = 0.49; Bonferroni corrected *p* = 0.033). The values 5 min after baseline and 30 min after baseline did not differ (1.74 ± 0.56 mA vs. 1.67 ± 0.58 mA; t(29) = 1.030; *p* = 0.321; Cohen’s d = 0.18; Bonferroni corrected *p* = 0.963). The results of the sensitivity measurements are depicted in Table 1 and Fig. 1.

**Table 1:** Results of the sensitivity threshold measurement.

	Total (n = 30)	Left side (n = 29)	Right side (*n* = *30*)
**Sensitivity threshold (mA; mean&SD)**			
Baseline	1.9 (± 0.59)	1.88 (± 0.66)	1.88 (± 0.6)
First value		2.07 (± 0.82)	2.05 (± 0.76)
Second value		1.81 (± 0.59)	1.91 (± 0.82)
Third value		1.78 (± 0.88)	1.85 (± 0.64)
5 min after baseline	1.74 (± 0.56)	1.69 (± 0.52)	1.78 (0.67)
30 min after baseline	1.67 (± 0.58)	1.61 (± 0.6)	1.7 (± 0.63)

The order of smell and taste examinations had no impact on the 30-min value of the sensitivity threshold (smell first: 1.63 mA ± 0.64 vs. taste first: 1.7 mA ± 0.53; *p* = 0.728; t-test).

## Discussion

Electrical threshold testing of the anterior faucial pillar is a simple, safe, and accurate diagnostic measure. By use of this method, we found a decrease over time in sensitivity threshold when the measurement was repeated 5 min and 30 min after baseline testing. A training effect seems unlikely, as the single values of the baseline measurement did not show this effect. The present data do not allow us to differentiate whether the lower sensitivity threshold 30 min after baseline is a prolonged facilitating effect of the ES or due to olfactory or gustatory information.

Stimulation of oropharyngeal structures with different modalities is used to improve the swallowing function of dysphagic patients. Olfactory information is thought to improve swallowing by activating the bilateral insular cortex, which is involved in central control of swallowing. Treating dysphagic patients with volatile black pepper oil improved their swallowing function^31^. Gustatory information is known to reduce the latency of the swallowing reflex. In particular, a sour bolus seems to have a large effect on the swallowing function^6,32^. Recently, the use of capsaicin has been evaluated for treating dysphagia. Capsaicin was found to increase substance P in the saliva. Substance P is a neuropeptide that enhances the swallowing and cough reflex^34^. Using capsaicin, Cabib et al. demonstrated a higher excitability of the motor cortex and enhancement of sensory conduction using transcranial magnetic stimulation^35^. Dysphagic patients showed an improvement in swallowing function when treated with capsaicin as compared to the placebo^36^. As olfactory and gustatory information has a positive impact on the swallowing function, it is conceivable that our test had an impact on the sensitivity threshold. With the present data, however, we are unable to distinguish between an effect of the ES or the taste and smell tests on the sensitivity threshold.

ES of oropharyngeal mucosa is an approved therapy for dysphagia following stroke. In theory, ES is thought to drive neuroplasticity by activating sensory fibres^37^. For pharyngeal electric stimulation (PES), an electrode mounted on a nasogastric tube is placed into the pharynx with contact to the posterior wall. The stimulation parameters with the largest effect were a stimulation at 5 Hz and a square wave duration of 200 µs with an individually adjusted stimulus intensity based on sensitivity threshold and maximum tolerated intensity^29,38,39^. PES was performed for 10 min on three consecutive days. Several smaller trials demonstrated a positive effect of PES on swallowing function^30,39,40^. However, a large multicentre trial failed to show this effect, which might be explained by the inclusion criteria and low stimulation intensity in the patients^41^. The same stimulation paradigm was used in a second multicentre trial in stroke patients tracheotomised due to dysphagia. This trial was terminated early because more patients receiving PES were ready for decannulation as compared to patients receiving sham stimulation^42^. ES of the faucial pillar region was also evaluated as a potential therapy method for dysphagia. Power et al. showed a facilitation of corticobulbar fibres using transcranial magnetic stimulation following a 10-min stimulation with a stimulus frequency of 0.2 Hz and a stimulus intensity of 75% of the maximum tolerated intensity^17^. When using this stimulation paradigm in dysphagic stroke patients, there was no effect on different swallowing parameters assessed by videofluoroscopy^43^. ES of the faucial pillar region has not been implemented into clinical routine. Our results might be explained by the effect of ES; however, our stimulation paradigm is different from the parameters that were used in prior experiments. Verifying our results in dysphagic patients regarding an improvement of swallowing function (using videofluoroscopy or flexible endoscopy of swallowing) would also be necessary to test for a clinical effect.

Thermal stimulation of oropharyngeal structures with ice (thermal-tactile stimulation = TTS) is a widely used approach in dysphagia therapy. This method is used to treat patients with an absent or delayed pharyngeal swallowing reflex. TTS is thought to improve the swallowing function by modulating the swallowing reflex. Alternatively, TTS may act as a heightened sensory input to central regions, facilitating a more rapid swallowing trigger^44^. An immediate effect of the swallowing function, such as increased speed of the swallowing trigger, has been described previously^20,21^. A review by Schwarz et al. did not show any evidence for a long-term effect on patients’ outcomes. The data on this matter must be interpreted carefully, as Schwarz et al. found large heterogeneity of the trials^44^.

As TTS and ES both include a mechanical component, the effect of mechanical stimulation has also been investigated. Sciortino et al. and Kaatzke-McDonald et al. found no effect of tactile stimulation on swallowing latency, using a probe to stroke the faucial pillar region^45,46^. As literature on the effect of pure tactile or mechanical stimulation in swallowing is sparse, the possibility remains that our results might at least partially be explained by the mechanical effect of the stimulation.

This study is limited by our inability to differentiate between an ongoing effect of the ES or an effect of the taste and smell tests, as described above. In case of an independent effect by smell or taste testing, it is not possible to determine which test is responsible for the effect. Repeating the test without sensoric testing or after a longer period (i.e., 24 h later) would help to further investigate the facilitating effect on the sensitivity threshold by ES. We are also unable to differentiate between an effect of the mechanical stimulation by the pressure of the finger-mounted electrode and the ES itself on the sensitivity. Further testing of patients in a clinical context is necessary to evaluate the clinical relevance of our findings. As we performed this experiment only in subjects below the age of 60, subjects with age 60 and above should be included, as dysphagia-related diseases (i.e., stroke or Parkinson’s disease) occur mainly in the elderly.

The facilitating effect must be kept in mind when sensitivity of the oral cavity is measured repeatedly in clinical trials. Measuring the sensitivity threshold in a standardized manner, as described below, offers the possibility of an objective and quantifiable measurement method for oral sensitivity. If the sensitivity threshold is assessed in the context of a clinical trial with dysphagic patients, we recommend measuring it first to circumvent a potential facilitating effect. In bedside screening, comprehensive examination of swallowing by a speech and language pathologist and endoscopic or videofluoroscopic evaluation of swallowing, the patient is usually given some amount of food and liquid. This might lead to a facilitating effect on the threshold, altering the results. By postponing the endoscopic evaluation of swallowing past ES and by conducting a standardized water-swallow test 4 h prior to ES, we estimate that the facilitating effect on sensitivity threshold will not occur and that the generated data might remain unbiased.

## Methods

### Participants

The study was conducted in a large German university hospital. The participants were healthy and below the age of 60.

The volunteers were included in the trial after providing written informed consent. The exclusion criteria comprised of a history of dysphagia (i.e., Parkinson’s disease, prior stroke, COPD, ENT tumours, dementia, etc.), known disturbances of smell and taste, known allergies to odorants or flavourings, or an implanted electrical device (i.e., cardiac pacemaker).

For the data acquisition and the use of findings for scientific analyses, an ethical approval was obtained from the local ethical committee (Justus-Liebig University, protocol number 149/16). All methods were carried out in accordance with relevant guidelines and regulations for involving human participants in the study. All participants gave informed consent.

The study was prospectively registered at www.clinicaltrials.gov (NCT03240965; registered 07/08/2017).

### Sensitivity threshold

Sensitivity testing of the oral cavity was performed by measuring the sensitivity threshold of the faucial pillar, as described by Power et al.^17^. By this approach, we were able to acquire quantifiable and objectifiable measurements. A commercially available pudendal electrode (St Mark’s Pudendal Electrode, Natus Neurology Incorporated, Middleton, Wisconsin, USA) was used for stimulation. Delivering the stimulation to the wet mucosa of the oral cavity, does not pose much of a problem, as the pudendal electrode (designed for transvaginal or transrectal stimulation of the pudendal nerve) is designed for such an environment. The adhesive electrode was mounted on the finger, with the anode (diameter 6 mm) attached directly on the fingertip. The stimulation occurs directly underneath the anode. The anode was placed with moderate pressure on the medial part of the anterior faucial pillar in the lower third just above the height of the last molar tooth. The cathode (diameter 3 mm) had contact with the buccal mucosa. During stimulation, the position of the fingertip was visually controlled.

The pudendal electrode was connected to a common electroneurography device (Dantec Keypoint, Natus Neurology Incorporated, Middleton, Wisconsin, USA). The electric stimuli were delivered with the continuous stimulation setting at a frequency of 3 Hz with a square wave duration of 200 ms. We increased the stimulus intensity in 0.2 mA steps until the participant felt the stimulus. The measurement was repeated three times for each side in random order. We calculated the mean for each side, as well as the mean of both sides taken together.

### Taste

To assess taste, we used a commercially available taste test kit (“Taste Strips”, Burghart Messtechnik, Wedel, Germany). This test was validated in a large study by Landis et al.^47^. A filter paper strip with a 2 cm^2^ tip area impregnated with different tastants (4 basic qualities in 4 different concentrations) was placed in the middle of the volunteer’s tongue. The taste strips featured the following concentrations: sweet: 0.4, 0.2, 0.1, 0.05 g/ml sucrose; sour: 0.3, 0.165, 0.09, 0.05 g/ml citric acid; salty: 0.25, 0.1, 0.04, 0.016 g/ml sodium chloride; and bitter: 0.006, 0.0024, 0.0009, 0.0004 g/ml quinine hydrochloride. A taste score was calculated from the number of correct answers.

### Smell

Smell was tested with a commercially available screening test using 12 different felt-tip pens soaked with different odorants (“Sniffin Sticks,” Burghart Messtechnik, Wedel, Germany). Each odorant was presented to the middle of the participant’s nose. The test contained aromatic and trigeminal odorants. For each odorant, the participant had to choose the correct answer from a list of four different options. A smell score was calculated from the number of correct answers^48^.

### Experimental procedure

**Figure 2:**
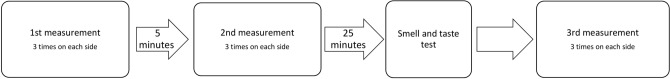
Workflow of the experiment.

Prior to testing, the participants fasted for at least one hour. The sensitivity was measured twice with a 5-min interval to test for facilitation of the threshold by the test procedure itself. Twenty-five minutes after the second measurement, we conducted the taste and smell tests to assess facilitation of the sensitivity threshold by those stimuli. The order of tests (smell first or taste first) was randomised. Afterwards, sensitivity was measured a third time. The workflow of the experiment is depicted in Fig. 2.

### Statistical analysis

Data are presented as mean and standard deviation. A repeated measures ANOVA was used to test for differences between the groups. For post-hoc tests, we used paired t-tests with correction for multiple comparisons (Bonferroni). For the remaining test, we used Student’s t-test. We also calculated Cohen’s d for the effect size. All statistical analyses were performed with SPSS, version 23.0 (©SPSS, Inc., IBM Company, 2015, Chicago, IL).

### Ethics approval and consent to participate

For the data acquisition and the use of findings for scientific analyses, an ethical approval was obtained from the local ethical committee (Justus-Liebig University, protocol number 149/16). All methods were carried out in accordance with relevant guidelines and regulations for involving human participants in the study. All participants gave informed consent.

## Data Availability

The authors declare that the data supporting the findings of this study are available within the article.

## Abbreviations

ES: Electrical stimulation
PES: Pharyngeal electrical stimulation
TTS: Thermal-tactile stimulation

## References

[1] Arnold M, Liesirova K, Broeg-Morvay A (2016). Dysphagia in acute stroke: incidence, burden and impact on clinical outcome. PLoS ONE.

[2] Warnecke T, Ritter MA, Kroger B (2009). Fiberoptic endoscopic Dysphagia severity scale predicts outcome after acute stroke. Cerebrovasc. Dis..

[3] Smithard DG, Smeeton NC, Wolfe CDA (2007). Long-term outcome after stroke: does dysphagia matter?. Age Ageing.

[4] Martino R, Foley N, Bhogal S (2005). Dysphagia after stroke: incidence, diagnosis, and pulmonary complications. Stroke.

[5] Heuschmann PU, Kolominsky-Rabas PL, Misselwitz B (2004). Predictors of in-hospital mortality and attributable risks of death after ischemic stroke: the German Stroke Registers Study Group. Arch. Intern. Med..

[6] Steele CM, Miller AJ (2010). Sensory input pathways and mechanisms in swallowing: a review. Dysphagia.

[7] Ertekin C (2011). Voluntary versus spontaneous swallowing in man. Dysphagia.

[8] Costa MMB (2018). Neural control of swallowing. Arq. Gastroenterol..

[9] Aida S, Takeishi R, Magara J (2015). Peripheral and central control of swallowing initiation in healthy humans. Physiol Behav.

[10] Ertekin C, Kiylioglu N, Tarlaci S (2001). Voluntary and reflex influences on the initiation of swallowing reflex in man. Dysphagia.

[11] Aviv JE (1997). Sensory discrimination in the larynx and hypopharynx. Otolaryngol. Head Neck. Surg..

[12] Aviv JE, Martin JH, Sacco RL (1996). Supraglottic and pharyngeal sensory abnormalities in stroke patients with dysphagia. Ann. Otol. Rhinol. Laryngol..

[13] Dziewas R, Warnecke T, Olenberg S (2008). Towards a basic endoscopic assessment of swallowing in acute stroke—development and evaluation of a simple dysphagia score. Cerebrovasc. Dis..

[14] Sellars C, Campbell AM, Stott DJ (1999). Swallowing abnormalities after acute stroke: a case control study. Dysphagia.

[15] Schimmel M, Voegeli G, Duvernay E (2017). Oral tactile sensitivity and masticatory performance are impaired in stroke patients. J. Oral. Rehabil..

[16] Theurer JA, Czachorowski KA, Martin LP (2009). Effects of oropharyngeal air-pulse stimulation on swallowing in healthy older adults. Dysphagia.

[17] Power M, Fraser C, Hobson A (2004). Changes in pharyngeal corticobulbar excitability and swallowing behavior after oral stimulation. Am. J. Physiol..

[18] Power ML, Hamdy S, Singh S (2007). Deglutitive laryngeal closure in stroke patients. J. Neurol. Neurosurg. Psychiatry.

[19] Prosiegel M, Heintze M, Wagner-Sonntag E (2002). Schluckstörungen bei neurologischen Patienten. Eine prospektive Studie zu Diagnostik, Störungsmustern, Therapie und Outcome. Nervenarzt.

[20] de Lama Lazzara G, Lazarus C, Logemann JA (1986). Impact of thermal stimulation on the triggering of the swallowing reflex. Dysphagia.

[21] Rosenbek JC, Robbins J, Fishback B (1991). Effects of thermal application on dysphagia after stroke. J. Speech Hear Res..

[22] Rosenbek JC, Roecker EB, Wood JL (1996). Thermal application reduces the duration of stage transition in dysphagia after stroke. Dysphagia.

[23] Ali GN, Laundl TM, Wallace KL (1996). Influence of cold stimulation on the normal pharyngeal swallow response. Dysphagia.

[24] Ali GN, Laundl TM, Wallace KL (1994). Influence of mucosal receptors on deglutitive regulation of pharyngeal and upper esophageal sphincter function. Am. J. Physiol..

[25] Veis SL, Logemann JA (1985). Swallowing disorders in persons with cerebrovascular accident. Arch. Phys. Med. Rehabil..

[26] Robbins J, Hamilton JW, Lof GL (1992). Oropharyngeal swallowing in normal adults of different ages. Gastroenterology.

[27] Suntrup S, Teismann I, Wollbrink A (2014). Pharyngeal electrical stimulation can modulate swallowing in cortical processing and behaviour—magnetoencephalographic evidence. Neuroimage.

[28] Hamdy S, Rothwell JC, Aziz Q (1998). Long-term reorganization of human motor cortex driven by short-term sensory stimulation. Nat. Neurosci..

[29] Fraser C, Power M, Hamdy S (2002). Driving plasticity in human adult motor cortex is associated with improved motor function after brain injury. Neuron.

[30] Scutt P, Lee HS, Hamdy S (2015). pharyngeal electrical stimulation for treatment of poststroke dysphagia: individual patient data meta-analysis of randomised controlled trials. Stroke Res. Treat..

[31] Ebihara T, Ebihara S, Maruyama M (2006). A randomized trial of olfactory stimulation using black pepper oil in older people with swallowing dysfunction. J. Am. Geriatr. Soc..

[32] Gatto AR, Cola PC, Da Silva RG (2013). Sour taste and cold temperature in the oral phase of swallowing in patients after stroke. Codas.

[33] Leopold NA, Kagel MC (1997). Dysphagia—ingestion or deglutition?: a proposed paradigm. Dysphagia.

[34] Suntrup-Krueger S, Muhle P, Kampe I (2021). Effect of capsaicinoids on neurophysiological, biochemical, and mechanical parameters of swallowing function. Neurotherapeutics.

[35] Cabib C, Nascimento W, Rofes L (2020). Short-term neurophysiological effects of sensory pathway neurorehabilitation strategies on chronic poststroke oropharyngeal dysphagia. Neurogastroenterol. Motil..

[36] Wang Z, Wu L, Fang Q (2019). Effects of capsaicin on swallowing function in stroke patients with dysphagia: a randomized controlled trial. J. Stroke Cerebrovasc. Dis..

[37] Cheng I, Sasegbon A, Hamdy S (2020). Effects of neurostimulation on poststroke dysphagia: a synthesis of current evidence from randomized controlled trials. Neuromodulation.

[38] Jefferson S, Mistry S, Michou E (2009). Reversal of a virtual lesion in human pharyngeal motor cortex by high frequency contralesional brain stimulation. Gastroenterology.

[39] Jayasekeran V, Singh S, Tyrrell P (2010). Adjunctive functional pharyngeal electrical stimulation reverses swallowing disability after brain lesions. Gastroenterology.

[40] Vasant DH, Michou E, O'Leary N (2016). Pharyngeal electrical stimulation in dysphagia poststroke: a prospective, randomized single-blinded interventional study. Neurorehabil. Neural Rep..

[41] Bath PM, Scutt P, Love J (2016). Pharyngeal electrical stimulation for treatment of dysphagia in subacute stroke: a randomized controlled trial. Stroke.

[42] Dziewas R, Stellato R, van der Tweel I (2018). Pharyngeal electrical stimulation for early decannulation in tracheotomised patients with neurogenic dysphagia after stroke (PHAST-TRAC): a prospective, single-blinded, randomised trial. Lancet Neurol..

[43] Power ML, Fraser CH, Hobson A (2006). Evaluating oral stimulation as a treatment for dysphagia after stroke. Dysphagia.

[44] Schwarz M, Ward EC, Ross J (2018). Impact of thermo-tactile stimulation on the speed and efficiency of swallowing: a systematic review. Int. J. Lang. Commun. Disord..

[45] Sciortino K, Liss JM, Case JL (2003). Effects of mechanical, cold, gustatory, and combined stimulation to the human anterior faucial pillars. Dysphagia.

[46] Kaatzke-McDonald MN, Post E, Davis PJ (1996). The effects of cold, touch, and chemical stimulation of the anterior faucial pillar on human swallowing. Dysphagia.

[47] Landis BN, Welge-Luessen A, Brämerson A (2009). "Taste Strips"—a rapid, lateralized, gustatory bedside identification test based on impregnated filter papers. J. Neurol..

[48] Hummel T, Sekinger B, Wolf SR (1997). ‘Sniffin’ sticks’: olfactory performance assessed by the combined testing of odor identification, odor discrimination and olfactory threshold. Chem. Senses.

